# Coronary Artery Bypass Grafting Versus Percutaneous Coronary Intervention for Multivessel Coronary Artery Disease: A One-Stage Meta-Analysis

**DOI:** 10.3389/fcvm.2022.822228

**Published:** 2022-03-25

**Authors:** Nicholas W. S. Chew, Jin Hean Koh, Cheng Han Ng, Darren Jun Hao Tan, Jie Ning Yong, Chaoxing Lin, Oliver Zi-Hern Lim, Yip Han Chin, Denzel Ming Wei Lim, Koo Hui Chan, Poay-Huan Loh, Adrian Low, Chi-Hang Lee, Huay-Cheem Tan, Mark Chan

**Affiliations:** ^1^Department of Cardiology, National University Heart Centre, Singapore, Singapore; ^2^Yong Loo Lin School of Medicine, National University of Singapore, Singapore, Singapore

**Keywords:** multivessel coronary artery disease (MVD), coronary artery bypass grafting (CABG), percutaneous coronary intervention, mortality, coronary artery

## Abstract

**Background and Aims:**

Data are emerging on 10-year mortality comparing coronary artery bypass grafting (CABG) and percutaneous coronary intervention (PCI) with stenting for multivessel disease (MVD) without left main (LM) involvement. We conducted an updated two-stage meta-analysis using reconstructed individual patient data to compare long-term mortality between CABG and PCI for patients with MVD without significant LM coronary disease.

**Methods:**

Medline and Embase databases were searched for articles comparing CABG with PCI for MVD. A two-stage meta-analysis was conducted using reconstructed patient level survival data for all-cause mortality with subgroups by SYNTAX score. The shared-frailty and stratified Cox models were fitted to compare survival endpoints.

**Results:**

We screened 1,496 studies and included six randomized controlled trials with 7,181 patients. PCI was associated with greater 10-year all-cause mortality risk (HR: 1.282, CI: 1.118–1.469, *p* < 0.001) compared with CABG. In patients with low SYNTAX score, 10-year all-cause mortality after PCI was comparable to CABG (HR: 1.102, 0.822–1.479, *p* = 0.516). However, in patients with moderate to high SYNTAX score, 10-year all-cause mortality was significantly higher after PCI compared with CABG (HR: 1.444, 1.122–1.858, *p* < 0.001; HR: 1.856, 1.380–2.497, *p* < 0.001, respectively).

**Conclusion:**

This updated reconstructed individual patient-data meta-analysis revealed a sustained lower cumulative all-cause mortality of CABG over PCI for multivessel disease without LM involvement.

## Introduction

Coronary artery disease (CAD) remains the leading cause of death worldwide (1). Current evidence has shown that coronary artery bypass grafting (CABG) increases survival in patients with left main (LM) coronary involvement over percutaneous coronary intervention (PCI) (2). There remains, however, the question of optimal revascularization strategy in patients with multivessel coronary artery disease (MVD) without LM coronary disease. When deciding between CABG and PCI for the revascularization of MVD, long-term mortality remains a key outcome of interest.

Previous meta-analyses comparing survival outcomes between PCI and CABG for MVD have utilized a standard meta-analytical approach (3–5). Head et al. performed an individual patient-data meta-analysis that demonstrated CABG having mortality benefit over PCI in patients with MVD, particularly those with diabetes and more complex coronary anatomy, with all-cause mortality reported only up to 5 years (2). Randomized controlled trials (RCTs) comparing PCI and CABG in patients with MVD have reported updated longer term findings, (6–11) with several studies reporting 10-year outcomes (9, 10). Hence, by including these RCTs with longer-term follow-up outcomes and adopting a two-stage meta-analysis using reconstructed individual patient data, this study sought to provide insights into the cumulative 10-year all-cause mortality following percutaneous and surgical coronary revascularization (12, 13).

## Methods

### Search Strategy and Selection Criteria

With reference to the Preferred Reporting Items for Systematic Review and Meta-Analyses (PRISMA) guidelines (14), a search was conducted on Medline and Embase databases for RCTs without language restriction relating to CABG and PCI for the treatment of MVD from inception to 23 July 2021. The search strategy used included terms “PCI,” “CABG,” and “RCTs.” The full search strategy is included in Supplementary Material 1. The references of included articles were also screened manually for a comprehensive search. We excluded trials enrolling patients with significant LM coronary artery stenosis, to reduce potential bias due to the presence of unprotected LM disease, as this was independently associated with increased morbidity and mortality among patients with CAD (15).

### Outcomes and Extraction

The primary outcome of the meta-analysis was all cause mortality. The secondary outcomes of the meta-analysis were repeat revascularization and myocardial infarction (MI). All-cause mortality was defined as any death events after intervention. MI included both procedural and non-procedural infarctions. Subgroup analyses were also performed based on the Synergy between PCI with Taxus and Cardiac Surgery (SYNTAX) scores, and stratified into low (0–22), moderate (23–32), and severe risk (>32).

The present RCTs have been limited by the lack of power to achieve significance, and often adopt the composite endpoint of major cerebrovascular and cardiac adverse events. Although composite outcomes may be helpful in allowing the individual studies to achieve statistically and clinically significant results, it limits the ability of the individual study to detect differences within each individual clinical outcome (16). Therefore, this timely study allows for the pooled analysis of hard endpoints such as all-cause mortality and attempts to mitigate the power limitations faced by the individual trials. Compared to traditional meta-analysis that apply a summary statistic for each individual study by simply assigning weight based on study size, the patient level meta-analysis avoids biases associated with the use of summary statistics by enabling the reanalysis of individual patients’ data to perform time-to-event analyses, and this has been widely considered to be the gold standard (13).

Three reviewers (CN, CHN, and JK) independently extracted the baseline characteristics and outcome measure in blinded pairs. To obtain individual patient data, Kaplan–Meier curves from the individual studies was performed using the methods of Guyot et al. (17). Briefly, this method entails downloading, pre-processing, and digitizing vector and raster images of survival or failure curves, such that their step function values, and corresponding timings can be obtained. This method is widely used in the reconstruction of data for individual patient data meta-analysis (18, 19). The extracted data from the respective Kaplan–Meier curves was conducted Web Plot Digitizer (20) with supplemented from the risk table to improve the calibration of extraction. Survival information on individual patients was recuperated by solving the inverted Kaplan–Meier product-limit equations (17, 20, 21). In the event of multiple publications of using the same cohort, studies which provided the most recent up to date evidence was used for the analysis. When the reconstruction of individual data was not feasible, we used a conventional two-stage approached to summarize the evidence.

### Risk of Bias Assessment

Two reviewers (CNH and JK) independently assessed the risk of bias of included RCTs using the Cochrane Risk-of-Bias tool (22), which evaluates seven domains including random sequence generation, allocation concealment, masking of participants and personnel, blinding of outcome assessment, incomplete outcome data, selective outcome reporting, and other sources of bias. Disagreements were resolved by consensus or appeal to a third author (CN).

### Statistical Analysis

All analysis was conducted in RStudio (Version 4.1.0) and Stata (16.1 StataCorp). The statistical analysis was conducted with a two-stage meta-analysis in hazard ratios (HR) for all-cause mortality with visual representation from a Kaplan–Meier curve. The primary analysis was conducted with a conventional frequential shared frailty model in gamma distribution which best accounts for study heterogenicity and assumes that individual patients within each study are similarly failure-prone as other individuals within that study (18, 19, 23–25). For each individual patient data analysis, we conducted three sensitivity analysis (1) Bayesian shared frailty, (2) stratified Cox regression, and (3) conventional two-stage analysis. The Bayesian shared frailty model was used as it most explicitly accounts for between-study heterogeneity *via* the involvement of random-effects term that models patients within the individual studies as being similarly failure-prone as other subjects within the same study (23–25). The Bayesian shared frailty analysis was conducted with 5,000 burns in and 50,000 iterations using the Jeffrey’s prior. Frailties are gamma-distributed across the articles, and affect the hazard function in a latent and multiplicative fashion. The output of the Bayesian analysis was HR and credible intervals (crl). As an additional sensitivity analysis, we used a stratified Cox regression with 10,000 bootstrapping of variance to reduce bias (18, 19). Proportionality in Cox regression was analyzed with the Grambsch–Therneau test for a non-zero slope and Schoenfeld residuals plot and no assumptions of proportionality was violated. Inter-study heterogeneity can be adjusted by Cox models through methods that allow subjects from the individual study to have a baseline hazard unique only to that study, while constraining partial likelihood estimates of the Cox coefficients to be equal across strata (23–26). When a one stage model was not feasible due to the lack of data (SYNTAX score subgroup for all-cause mortality and MI), a conventional two-stage analysis with inverse variance weighting was conducted in fixed and random effects (12) to pool the summary HR of individual trials. The computed two-stage HRs allows for correction for publication bias and small-study effects *via* the random effects trim-and-fill (R0 estimator) procedure. Statistical significance was considered for outcomes with a *p* value ≤ 0.05. Statistical heterogeneity was assessed *via* I^2^ and Cochran *Q* test values (27), where an *I*^2^ value of 25, 50, and 75% represented low, moderate, and high degree of heterogeneity, respectively (28, 29). A Cochran *Q* test with *p*-value of ≤0.10 was considered significant for heterogeneity.

## Results

### Summary of Included Articles

**FIGURE 1:**
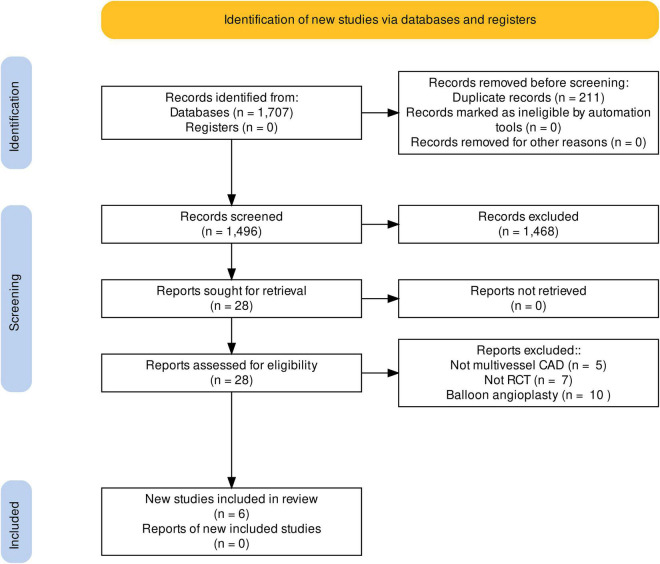
PRISMA flow diagram. Search strategy and source of included studies.

**FIGURE 2:**
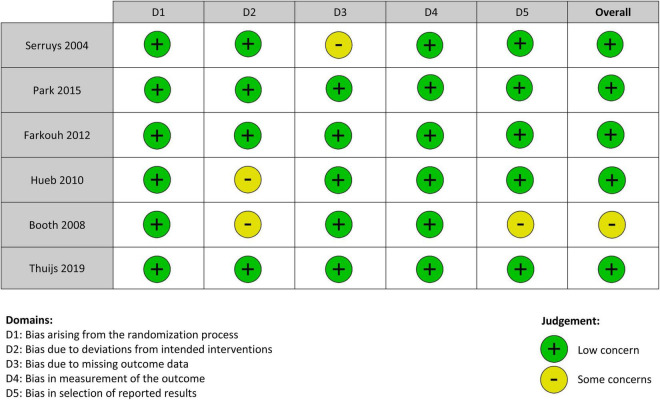
Risk of bias assessment.

Of the total of 1,496 articles included in the initial search after removal of duplicates, 28 were selected for full text review. Six RCTs met the final inclusion criteria (Figure 1). There were 7,181 patients in total, of whom 3,587 were assigned to the PCI group and 3,594 to the CABG group. The mean age of patients was 63 years, and the median follow-up was 5 years. All studies were assessed using the Cochrane Collaboration’s Risk of Bias Assessment tool (22). Five studies were judged to be of low risk of bias (6–10) and one study was assessed to be at moderate risk of bias (11) (Figure 2). One trial was a single-center study conducted in Brazil (10). The other five trials were multicenter trials conducted across the Netherlands, Brazil, United Kingdom, United States, Austria, South Korea, Thailand, Malaysia, China, Canada, Italy, Switzerland, France, Germany, Poland, Spain, Czechia, Israel, Argentina, New Zealand, Australia, India, Sweden, Finland, Belgium, Norway, Denmark, and Portugal (6–8, 11, 30). Three RCTs used bare-metal stents (9–11), one used paclitaxel drug eluting stents (DES) (8), one used everolimus DES (6), and one used paclitaxel or sirolimus DES (7). A summary of the characteristics of included studies is provided in Table 1.

**TABLE 1:** Characteristics of included studies.

Study	PCI (n)	CABG (n)	Country	Male (%)	Smoker (%)	Diabetes (%)	Hypertension (%)	Unstable angina (%)	Previous MI (%)	Mean age (years)	Mean SYNTAX score	Type of stent used	Mean follow-up (years)	Mean LV ejection fraction (%)
ARTS	600	605	Netherlands, Brazil, United Kingdom, United States, Austria	76.5	27.0	17.5	45.0	37.0	44.0	60.6 ± 10.8		Bare metal stent	4.8 ± 0.9	60.5 (12.5)
BEST	438	442	South Korea, United States, Thailand, Malaysia, China	71.5	20.1	41.3	67.1	42.2	9.8	64.5 ± 9.4	24.4 (7.8)	Everolimus DES	4.0 ± 1.3	59.5 (8.3)
FREEDOM	953	947	United States, Canada, Italy, United Kingdom, Switzerland, France, Germany, Poland, Spain, Czechia, Israel, Brazil, Argentina, New Zealand, Australia, India	71.4	15.7	100	85.0	31.0	26.2	62.1 ± 9.1	26.2 (8.6)	Paclitaxel and sirolimus DES	3.5 ± 1.4	66.1 (11.3)
MASS-II	205	203	Brazil	69.5	29.5	26.0	62.0	0	52.0	59.8 ± 9.0		Bare metal stent	4.5 ± 1.3	67 (8.5)
SoS	488	500	United Kingdom, Sweden, Finland, Spain, Belgium, Italy, Canada, Germany, Switzerland, Norway	79.0	15.0	14.5	45.0	20.0	44.0	61.4 ± 9.3		Bare metal stent	4.7 ± 0.9	57.0
SYNTAX	903	897	Netherlands, Germany, United Kingdom, United States, France, Denmark, Spain, Belgium, Italy, Hungary, Sweden, Finland, Portugal, Czechia, Austria	77.5	20.0	25.5	66.5	29.0	32.0	65.1 ± 9.7	28.7 (11.5)	Paclitaxel DES	4.4 ± 1.4	

*PCI, percutaneous coronary intervention; CABG, coronary artery bypass grafting; MI, myocardial infarction; DES, drug eluting stent; LV, left ventricle.*

### All-Cause Mortality

**FIGURE 3:**
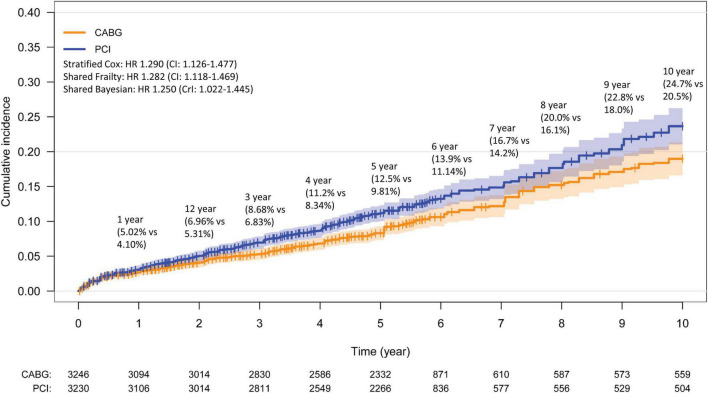
Kaplan–Meier curve for all-cause mortality. Kaplan–Meier estimates for all-cause mortality from the overall pooled patient population. CABG, coronary artery bypass graft; PCI, percutaneous coronary intervention.

There was a total of 3,230 and 3,246 patients treated with PCI and CABG with a mean survival time of 5.4 and 5.4 years, respectively. The mean follow up time was 4.3 years, with 2 trials providing 10-year follow-up data (8, 10), 1 with 6-year (11), and 3 with 5-year (6, 7, 9). There was a significant increase in all-cause mortality by 28.2% in the PCI arm compared to the CABG arm using the one stage shared frailty model (HR: 1.282, CI: 1.118–1.469, *p* < 0.001). Sensitivity analysis using a Bayesian shared frailty with uninformative priors yielded similar results (HR: 1.250, Crl: 1.022–1.445). The stratified Cox regression (HR: 1.290, CI: 1.126–1.477) yielded consistent results with the shared frailty models. In a two-stage analysis, both the fixed (HR: 1.292, CI: 1.123–1.486) and random effects (HR: 1.284, CI: 1.110–1.500) yielded significantly increase in all-cause mortality with PCI compared to CABG. The reconstructed 10-year Kaplan–Meier curve for all-cause mortality shows similar mortality following PCI and CABG within the first year, with gradual divergence of the time-to-event curves beyond 2 years (Figure 3). Low heterogeneity was detected in this analysis (I^2^: 14.4%; *p* = 0.322).

A subgroup analysis was performed based on the SYNTAX score. The mortality following PCI and CABG among patients with low SYNTAX risk was not significantly different (HR: 1.102, 0.822–1.479, *p* = 0.516). Heterogeneity detected in this analysis was low (*I*^2^ = 0%, *p* = 0.950). However, patients with moderate and severe SYNTAX score were found to have increased all-cause mortality following PCI when compared to CABG (HR: 1.444, 1.122–1.858, *p* < 0.001; HR: 1.856, 1.380–2.497, *p* < 0.001, respectively) with low heterogeneity (*I*^2^ = 0%, *p* = 0.71, *I*^2^ = 0%, *p* = 0.540).

### Secondary Outcomes

Data on repeat revascularization were available for 1,982 and 1,958 patients treated with PCI and CABG, respectively. There was a significant increase in repeat revascularization following PCI when compared to CABG based on the shared frailty model (HR: 3.234, CI: 2.624–3.986, *p* < 0.001). Incidence of MI could be evaluated in 2,196 patients treated with PCI and 2,197 patients treated with CABG. MI estimates from the fixed effect showed a 67% increase in MI in the PCI arm compared to the CABG arm (HR: 1.675, 1.362–2.060, *p* < 0.001) with the random effects showing similar estimates (HR: 1.662, 1.297–2.129, *p* < 0.001). A summary of the results for revascularization and MI are found in Supplementary Table 1.

## Discussion

This meta-analysis of six RCTs compares longer-term outcomes of PCI with stents versus CABG for the treatment of MVD without LM CAD (31–34). Building on the previous findings by Head et al. (2), we provide an updated analysis of 10-year all-cause mortality using reconstructed individual patient data. Our analysis found that the 10-year all-cause mortality was higher in those who received PCI arm compared CABG arm among 7,181 patients with MVD. Subgroup analyses by SYNTAX score showed that PCI was associated with significantly higher all-cause mortality compared to CABG in patients with moderate to high SYNTAX scores, but conferred similar survival benefit to CABG in patients of low SYNTAX score. Moreover, CABG remains the ideal revascularization strategy in MVD conferring lower repeat revascularization and MI risk compared to the PCI treatment arm.

With the advancement of PCI including stent design, higher risk patients with more complex coronary lesions have been included in RCTs. As such, we have observed a larger relative 10-year mortality benefit of CABG over PCI in our study, particularly in those with more complex CAD. Our findings are in line with the shorter-term outcomes reported by previous meta-analysis (2) and large real-world propensity score matched registries (35, 36). We demonstrated, for the first time, that the cumulative all-cause mortality was similar between PCI and CABG in the initial few years following revascularization, but only became more apparent with larger mortality benefit in favor of CABG as follow-up time progressed to a longer term. Our findings add to the current knowledge that the favorable prognosis conferred by CABG was sustained for up to 10 years. Even though the lack of data prevented subgroup analysis with other important clinical factors such as diabetes (4) and sex (37), our findings remain as important considerations when selecting the revascularization strategy of choice for patients with MVD.

The lower mortality with CABG compared to PCI is consistent with the SYNTAX trial that used first-generation (8) and the BEST trial that used second-generation DES (6). However, there is ongoing debate whether the relative benefits of CABG compared to PCI with DES are related with the improvements in stent designs. As such, with the advancement of stent designs, this led to the inclusion of higher-risk participants with increasing CAD complexity in recent RCTs. With recent evidence of the increased 5-year all-cause mortality in both CABG and PCI cohorts in the more recent trials with DES compared to previous trials with BMS (2), the benefit of CABG over PCI is more likely to be associated with the increasing inclusion of trial participants with more complex CAD.

A higher SYNTAX score has been associated with poorer prognosis in patients undergoing revascularization (38) and subgroup analysis found no significant difference between PCI and CABG in patients with low SYNTAX score. Current guidelines from the American Heart Association/American College of Cardiology (AHA/ACC) and European Society of Cardiology/European Society of Thoracic Surgeons (ESC/ESTS) guidelines recommend that low-complexity MVD can be treated with PCI, such as lesions without side branch involvement or without total occlusions, whereas relatively more complex MVD (intermediate or high SYNTAX score or triple vessel disease) is best treated with CABG (39, 40). However, PCI is often associated with lower rates of stroke and infection (41) with better patient acceptance. Data from contemporary landmark trials yielded mixed results with the SYNTAX trial (42) showing superiority of CABG over PCI in individuals with SYNTAX scores > 22, while the BEST (6) and FREEDOM (7) trials did not show any prognostic benefit of CABG over PCI irrespective of the SYNTAX score. With this large individual patient-data level meta-analysis, PCI confers similar long-term mortality benefit compared to CABG in a carefully selected group of patients, namely patients with low SYNTAX score and MVD without LM CAD (43).

One of the well-established benefits of CABG over PCI is its favorable incidence of repeat revascularization. The higher risk of repeat revascularization in the PCI group, compared to the CABG group, found in our study is consistent with the current literature (2). Observational data suggest good long-term of graft patency following CABG with 15-year patency of the left internal mammary artery of up to 95% (44), and 10-year patency of saphenous vein grafts of 86% (45). However, some of the available RCTs compared the newest generation of stents to the “old fashioned” grafting strategies (i.e., venous grafting) that had more limited durability (43). As such, futures RCTs comparing the utilization of newer stent technology and grafting strategies (that is, total arterial revascularization) might lead to more compelling results in the difference in revascularization rates. Moreover, the pooled analysis found a lower incidence of MI after CABG compared with PCI over time. CABG allows the advantage of addressing the overall burden of diffuse atherosclerotic disease by constructing the anastomosis distal to the stenotic segments, whilst PCI only addresses the flow-limiting stenoses without treating the distally stenosed lesions. CABG also allows for higher rates of complete revascularization, which is challenging for PCI in complex MVD (8). Additionally, the grafting of conduit vessels to the mid coronary vessels has been hypothesized to provide a protective effect against the development of new proximal coronary artery disease (1, 46, 47). Arterial grafts have also been demonstrated to release substantial amounts of nitric oxide, which is hypothesized to induce angiogenesis and improve the microvascular network of neo-capillaries within the myocardium (48–50). All these factors are likely to confer reduced mortality, MI and repeat revascularization risks in favor of CABG over PCI in patients with MVD.

### Clinical Implications

The ongoing debate between cardiologists and cardiac surgeons on revascularization strategy is partly due to the lack of long-term follow-up in comparative PCI and CABG trials, and this updated meta-analysis aims to fill such gap. The profound importance of heart team remains the core in deciding optimal revascularization strategy for patients with MVD in the absence of LM CAD. Current longer-term evidence from the latest RCTs suggests that CABG is preferred to PCI in cases when the SYNTAX score is >22 for survival benefit. Our findings also reinforce the favorable repeat revascularization and MI profile of CABG over PCI in patients with MVD.

### Strengths and Limitations

This meta-analysis used reconstructed individual patient data and rigorous statistical methods on 7,181 participants of RCTs for 10-year all-cause mortality. However, our study has the following limitations. First, in some of the RCTs (8), PCI was performed with first-generation drug-eluting stents that are no longer available. The use of newer-generation drug-eluting stents may provide significantly improved outcomes for patients, including reduction of mortality. Second, definitions of outcomes and patient characteristics might have varied between trials. However, measures of statistical heterogeneity were mostly low in majority of the analysis. Third, the RCTs assumed clinical equipoise between revascularization strategies. A proportion of patients might not be eligible for inclusion into the trials included in this analysis as some might have coronary lesions too complex for PCI treatment or have operative risks too high for CABG, thus rendering the results not generalizable to the entire population of individuals with MVD (2). Fourth, the outcome of stroke was not analyzed in this study due to the lack of available data. Future studies are warranted to evaluate stroke outcomes as they affect quality of life and morbidity. Heart teams should consider the optimal revascularization strategy based on the collective risk profile of MI, stroke, and repeat revascularization in addition to mortality. Fifth, most meta-analyses are limited by the relatively short follow-up time and unable to establish the full effect of revascularization strategy on long-term mortality. To date, this updated meta-analysis provides outcomes with the longest follow-up time. However, only 2 RCTs have reported 10-year outcomes (8, 10), which limited the statistical power of the study, and estimates over longer follow-up times may be less robust. Further RCTs with longer follow-up are needed to better examine the mortality difference between these revascularization strategies. Furthermore, although the algorithm applied for the reconstruction of individual patient data meta-analysis enabled close approximation to the original individual patient time-to-event within the matched studies, it did not have additional patient-level covariates that could potentially allow deeper insights into various subgroup analyses. Lastly, CARDia trial was not included as only the composite outcomes of death, stroke, MI and/or revascularization were presented in the survival analysis, and individual data was not available for the individual components of mortality, MI or stroke (51). On the other hand, VA CARDS and ERACI II trials were not included in the analysis due to the individual trial’s inclusion criteria that enrolled participants with isolated left anterior descending coronary disease as well as left main coronary disease, respectively (52, 53). Rather, a stringent inclusion criteria was adopted for the present analysis to avoid heterogeneity in the study population for the comparison of revascularization strategies in patients with MVD without LM CAD.

## Conclusion

In conclusion, this reconstructed individual patient data meta-analysis of 7,181 patients provides updated evidence on the significant mortality benefit of CABG over PCI in patients with MVD, extending up to 10-years of follow-up. This mortality benefit was observed particularly amongst patients with moderate to high SYNTAX score. However, PCI and CABG conferred similar survival benefit in patients with low SYNTAX score. Although the results match reasonably well with the current guideline recommendations, the study offers a robust and up-to-date appraisal of longer-term outcomes of the revascularization strategies with the inclusion of the recent 10-year trials. The longer term survival analysis on this important topic will facilitate the Heart team in making informed decisions in the outcomes of each revascularization strategy.

## Data Availability Statement

The original contributions presented in the study are included in the article/Supplementary Material, further inquiries can be directed to the corresponding authors.

## Author Contributions

JK, NC, and CN: conceptualization, data curation, and writing original draft. JK, NC, CN, DT, JY, CL, OL, YC, and DL: formal analysis. KC, P-HL, AL, C-HL, H-CT, and MC: supervision and validation. DT, JY, CL, OL, YC, DL, KC, P-HL, AL, C-HL, H-CT, and MC: writing, review, and editing. All authors have read and approved the final version of the manuscript for submission.

## Conflict of Interest

MC received speaker’s fees and research grants from Astra Zeneca, Abbott Technologies, and Boston Scientific. The remaining authors declare that the research was conducted in the absence of any commercial or financial relationships that could be construed as a potential conflict of interest.

## Publisher’s Note

All claims expressed in this article are solely those of the authors and do not necessarily represent those of their affiliated organizations, or those of the publisher, the editors and the reviewers. Any product that may be evaluated in this article, or claim that may be made by its manufacturer, is not guaranteed or endorsed by the publisher.
