# Supplementary material for: Coronary Artery Bypass Grafting Versus Percutaneous Coronary Intervention for Multivessel Coronary Artery Disease: A One-Stage Meta-Analysis
Source: Front Cardiovasc Med. 2022 Mar 25;9:822228. doi: 10.3389/fcvm.2022.822228 (PMC8990308; doi:10.3389/fcvm.2022.822228)
Supplement: Supplementary file 1 [file Data_Sheet_1.DOCX]

**Supplementary Material 1**

**Search strategy for Medline**

1 exp Percutaneous Coronary Intervention/ **or** pci.tw. **or** (percutaneous coronary adj3 (interven* **or** revascular*)).tw. **or** exp Stents/ **or** stent*.tw. **or** exp Drug-Eluting Stents/ **or** exp Angioplasty, Balloon, Coronary/ **or** (angioplast* adj3 coronary).tw. **or (**balloon angioplast*).tw. **or** (transluminal adj3 angioplast*).tw. **or** PTA.tw. **or** PTCA.tw.

2 exp Coronary Artery Bypass/ **or** cabg.tw. **or** (coronary adj3 bypass*).tw. **or** (aortocoronary adj3 bypass*).tw.

3 multivessel*.tw.

4 (clinical adj3 trial).tw. or (singl$ OR doubl$ OR trebl$ OR tripl$).tw. AND (mask$ OR blind$).tw. or placebo$.tw. or random$.tw. or exp randomized controlled trials/ or exp random allocation/ or exp double-blind method/ or exp single-blind method/ or exp placebos/ or research design/

5 1 and 2 and 3 and 4

6 Limit 5 to English

**Search strategy for Embase**

#1 pci:ti,ab or (percutaneous coronary NEXT/3 (interven* or revascular*)):ti,ab or stent*:ti,ab or (angioplast* NEXT/3 coronary):ti,ab or (balloon angioplast*):ti,ab or ((transluminal or trans‐luminal) NEXT/3 angioplast*):ti,ab or PTA.tw. or PTCA:ti,ab

#2 cabg:ti,ab **or** (coronary NEXT/3 bypass*):ti,ab **or** (aortocoronary NEXT/3 bypass*):ti,ab

#3 multivessel*:ti,ab

#4 crossover$:ti,ab or cross over$:ti,ab or placebo$:ti,ab or doubl$:ti,ab adj blind$:ti,ab or allocat$:ti,ab or random$:ti,ab or trial:ti,ab or ‘crossover-procedure’:ti,ab or ‘double-blind procedure’:ti,ab or ‘single-blind procedure’:ti,ab or ‘randomized controlled trial’:ti,ab or 'randomized controlled trial'/exp or 'controlled clinical trial'/exp

#5 #1 AND #2 AND #3 AND #4

#6 #5 AND [English]/lim
