# Supplementary material for: Coronary Artery Bypass Grafting Versus Percutaneous Coronary Intervention for Multivessel Coronary Artery Disease: A One-Stage Meta-Analysis
Source: Front Cardiovasc Med. 2022 Mar 25;9:822228. doi: 10.3389/fcvm.2022.822228 (PMC8990308; doi:10.3389/fcvm.2022.822228)
Supplement: Supplementary file 2 [file Table_1.docx]

**Supplementary Table 1: Summary of meta-analysis for myocardial infarction and repeat revascularization**

| **Events** | **Effect Size** | **p-value^*^** |
| --- | --- | --- |
| **Repeat Revascularisation** |  |  |
| One Stage Frailty | 3.234 (CI: 2.642 - 3.986) | <0.0001 |
| One Staged Bootstrapped^1^ | 3.195 (CI: 2.592 - 3.938) | <0.0001 |
| One Stage Bayesian Analysis | 3.227 (Crl: 2.648 - 4.022) | - |
| Two Stage (Fixed Effects) | 3.297 (CI: 2.753 - 3.949) | <0.0001 |
| Two Stage (Random Effects) | 3.231 (CI: 2.541 - 4.109) | <0.0001 |
|  |  |  |
| **Myocardial infarction** |  |  |
| Two Stage (Fixed Effects) | 1.675 (CI: 1.362-2.060) | <0.01 |
| Two Stage (Random Effects) | 1.662 (CI: 1.297-2.129) | <0.01 |

*Denotes p-value comparing HR of PCI versus CABG

^1^Denotes bootstrapping with 10,000 repetitions

CrI – credible interval, HR – hazard ratio, CI – confidence interval
